# Study on Selected Metal-Organic Framework-Based Catalysts for Cycloaddition Reaction of CO_2_ with Epoxides: A Highly Economic Solution for Carbon Capture and Utilization

**DOI:** 10.3390/polym13223905

**Published:** 2021-11-11

**Authors:** Suleiman Gani Musa, Zulkifli Merican Aljunid Merican, Omid Akbarzadeh

**Affiliations:** 1 Fundamental and Applied Sciences Department, Universiti Teknologi PETRONAS, Bandar Seri Iskandar 32610, Malaysia; ganisuleiman@gmail.com; 2 Department of Chemistry, Al-Qalam University Katsina, PMB 2137, Tafawa Balewa Way, Dutsin-ma Road, Katsina 820252, Nigeria; 3 Institute of Contaminant Management for Oil & Gas, Universiti Teknologi PETRONAS, Bandar Seri Iskandar 32610, Malaysia; 4 Nanotechnology & Catalysis Research Centre (NANOCAT), University of Malaya, Kuala Lumpur 50603, Malaysia; omid.akbarzadeh63@gmail.com

**Keywords:** cycloaddition, epoxides, carbon dioxide, metal-organic frameworks

## Abstract

The level of carbon dioxide in the atmosphere is growing rapidly due to fossil fuel combustion processes, heavy oil, coal, oil shelter, and exhausts from automobiles for energy generation, which lead to depletion of the ozone layer and consequently result in global warming. The realization of a carbon-neutral environment is the main focus of science and academic researchers of today. Several processes were employed to minimize carbon dioxide in the air, some of which include the utilization of non-fossil sources of energy like solar, nuclear, and biomass-based fuels. Consequently, these sources were reported to have a relatively high cost of production and maintenance. The applications of both homogeneous and heterogeneous processes in carbon capture and storage were investigated in recent years and the focus now is on the conversion of CO_2_ into useful chemicals and compounds. It was established that CO_2_ can undergo cycloaddition reaction with epoxides under the influence of special catalysts to give cyclic carbonates, which can be used as value-added chemicals at a different level of pharmaceutical and industrial applications. Among the various catalysts studied for this reaction, metal-organic frameworks are now on the frontline as a potential catalyst due to their special features and easy synthesis. Several metal-organic framework (MOF)-based catalysts were studied for their application in transforming CO_2_ to organic carbonates using epoxides. Here, we report some recent studies of porous MOF materials and an in-depth discussion of two repeatedly used metal-organic frameworks as a catalyst in the conversion of CO_2_ to organic carbonates.

## 1. Introduction

**Figure 1:**
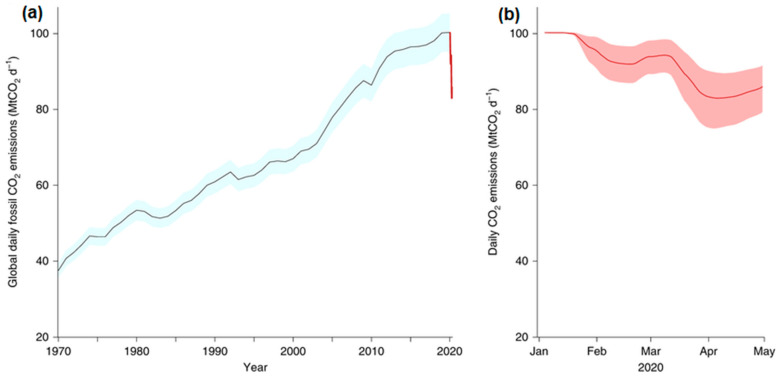
Daily global CO_2_ emissions: (**a**) Mean daily annual emissions of CO_2_ from 1970–2019 (**b**) Daily CO_2_ emissions decline in 2020 due to low anthropogenic activities. Reprinted with permission from Ref. [20]. Copyright 2020 Springer Nature.

The carbon dioxide (CO_2_) concentration in the atmosphere is becoming alarming due to an increase in anthropogenic activities such as fuel combustion and other energy generation processes (see Figure 1), which may result in global warming [1,2,3,4,5,6]. Carbon capture, storage, and utilization are the most promising alternative among other several processes in reducing CO_2_ emission [7,8,9]. The process of carbon capture and sequestration (CCS) was employed initially by using classical solid sorbents such as zeolites, porous carbon, porous silica, and mineral carbonation processes [10,11,12]. In recent years, the attention of the researchers has dwelled on utilizing CO_2_ as C_1_ feedstock for chemical conversion into various useful products [13,14,15] in the presence of highly selective and task-specific catalysts [8,16,17]. This route has a more beneficial advantage as it allows the utilization of unwanted substances into useful materials [18,19].

**Figure 2:**
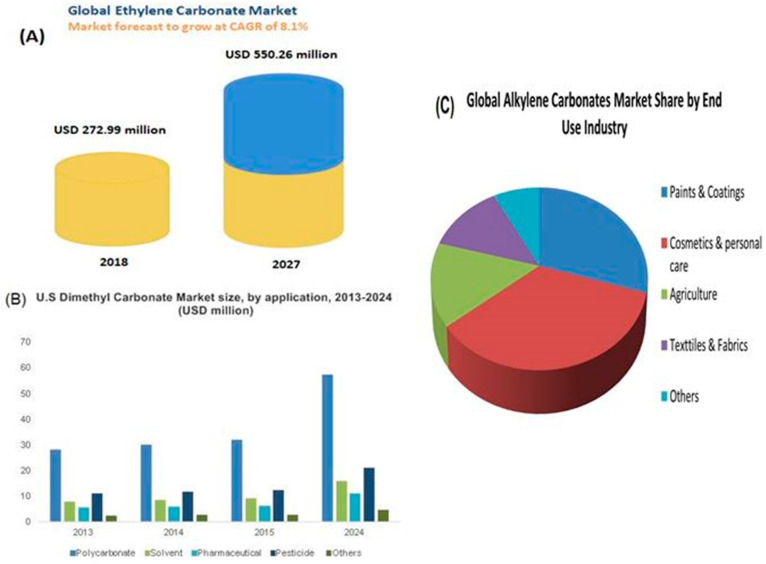
(**A**) Global ethylene carbonate forecast. (**B**) United States’ market growth by application for dimethyl carbonate. (**C**) The global alkylene carbonates market share by end use industry 2023 forecast [23].

One of the possible optimistic approaches to climate-relevant carbon capture and sequestration is the catalyst-mediated reaction of CO_2_. Industrial and atmospheric CO_2_ are captured and passed through biochemical, photochemical, thermochemical, or electrochemical conversion process to obtained intermediates such as methanol, dimethyl ether (DME), syngas, polyols, and cyclic organic carbonates, which can be further converted into end products as liquid fuels, aromatics among others, or used in the production of cosmetic and personal care products. The search for specific and specially designed catalysts for CO_2_ conversion into commercially relevant compounds is indeed a subject of high curiosity these days [16,21,22]. This review focused on the CO_2_ conversion by cycloaddition reactions with epoxides and the application of different polymeric metal-organic frameworks (MOFs) materials as potential catalysts that give an intermediate that can be converted into different organic chemicals such as diethyl carbonate (DEC), ethyl methyl carbonate (EMC), and dimethyl carbonate (DMC), among others. Over the years, there has been increasing demand for cyclic carbonate intermediates in various end-use industries such as electrolyte solvents in lithium-ion battery, medical, industrial, and personal care. For instance, according to the *Research and Markets* data statistic forecast report, the global ethylene carbonate market size alone was estimated to be valued at approximately USD 273 million in 2018 and projected to reach over USD 500 million by 2027, at a compound annual growth rate (CAGR) of 8.1% Figure 2A. The market size for dimethyl carbonate (DMC) was valued over USD 410 million in 2015 and was forecast to have gains exceeding 5% CAGR. In the United States of America (USA) alone, it was estimated to grow over USD 100 million by 2024 in its application as solvents, polycarbonates, and in pharmaceutical, and pesticide industries among others as forecast by *Global Market Insights*, as shown in Figure 2B. The global polycarbonate business was valued at over USD 14 billion in 2015 and was projected to reach USD 22 billion by 2024. Alkylene carbonates in general, which include, ethylene carbonate, propylene carbonate, glycerin carbonate, etc., are cyclic esters of carbonic acid that possess various properties such as low volatile organic content (VOC), low toxicity, being readily biodegradable, high boiling point, low-odor, and all these properties make them an excellent solvent choice. The global alkylene carbonates market share by end use industry (paints and coatings, agriculture, textiles and fabrics, cosmetics and personal care, and others) is shown in Figure 2C.

**Figure 3:**
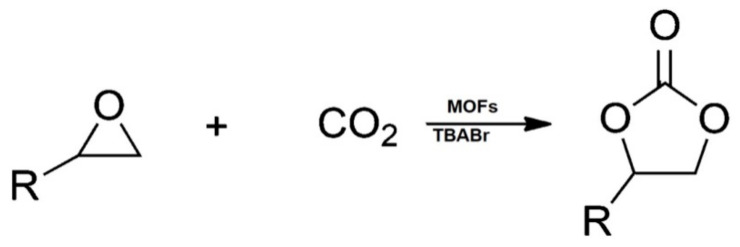
General scheme for cycloaddition reaction of CO_2_ and epoxide using metal-organic frameworks (MOF) catalysts and tetrabutylammonium bromide (TBABr) as co-catalyst.

The revolutionary work in the investigation of metal-organic frameworks (MOFs) as a catalyst for cycloaddition reaction was first discovered in 2009, where the renowned MOF-5 was discovered to have successfully catalyzed CO_2_ conversion reaction with epoxides in the presence of quaternary ammonium salts such as tetrabutylammonium bromide (TBABr), see (Figure 3). In the last decade, different catalysts were designed to facilitate CO_2_ conversion reaction into cyclic organic carbonates [24]. The features and composition of post-combustion CO_2_ indicated that abilities such as high CO_2_ uptake and selectivity, excellent thermal and chemical stability, good reusability, easy synthesis, and operation at ambient conditions should be given by an ideal adsorbent for better CO_2_ capture [22].

Catalytic materials such as amines [25,26], alkali metal salts [25,26,27,28,29,30,31], metal oxides [32,33], metal porphyrins [34,35,36], ionic liquids hybrids [37,38,39,40,41,42], zeolites [43,44,45,46,47], coordination metal complexes [47,48,49,50,51], and metal-organic frameworks [5,13,52,53,54,55,56,57,58,59,60,61,62,63,64,65] were reported recently to catalyze the formation of cyclic carbonates from CO_2_ [66,67,68,69,70,71,72,73,74,75,76,77,78,79,80]. Transition metal ion-based catalysts in conjunction with Lewis-base were also reported as a potential catalyst for the reaction [56,81,82,83]. Although certain conversion and selectivity were achieved, yet many of the reported catalytic system had shown one of the following disadvantages: need for high concentration of catalyst, instability of catalyst, need for co-catalyst, requirement of higher temperature and pressure, longer duration for the completion of reaction, or difficulty in separating the catalyst after the reaction for reuse [84]. Among the different porous materials reported, MIL-101(Cr) and Cu-BTC were among the few MOFs that showed resistance to many of the problems defeating other MOF materials [85,86,87,88].

## 2. Reaction Mechanism for the Production of Cyclic Carbonates from CO_2_ and Epoxides

CO_2_ conversion into cyclic carbonate compounds by cycloaddition reaction to epoxide is regarded as a method with economic advantages to attain a CO_2_-neutral environment and to serve as a source for value-added chemicals. Various researchers reviewed the mechanism for cycloadding CO_2_ into epoxides [8,14,17,65,89,90,91,92].

**Figure 4:**
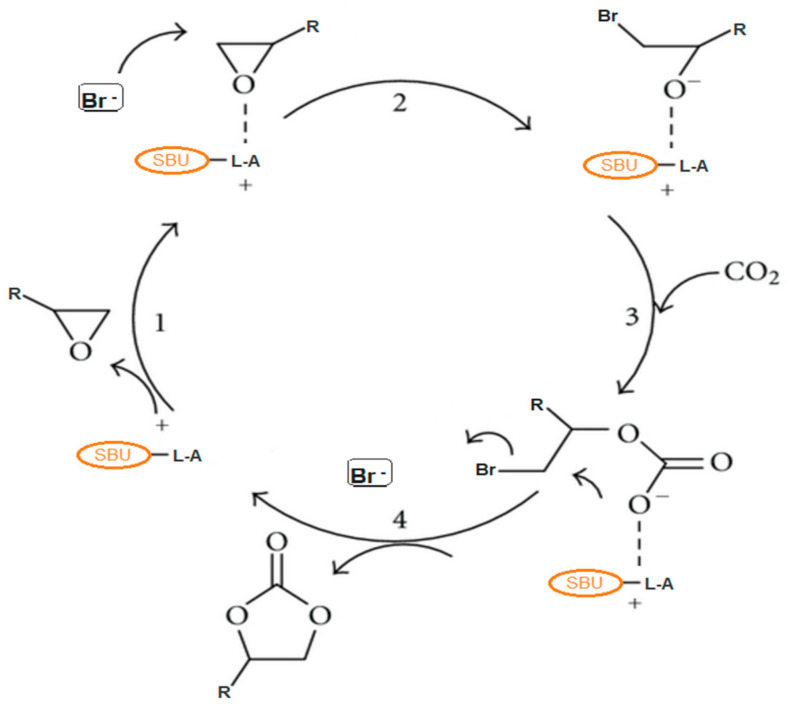
The general schematic reaction mechanism for CO_2_ cycloaddition with epoxides catalyzed by a Lewis-acid catalyst MOF and TBABr co-catalyst presence.

The process requires a robust acid catalyst to stimulate the epoxide substrate and the highly stable CO_2_ double bond and thermodynamically facilitates the opening of the epoxide ring via nucleophilic co-catalyst (TBABr) attack forming an alkoxide as an intermediate, which subsequently combines with the CO_2_-adduct to give the desired carbonates (Figure 4). The TBABr co-catalyst functions as a nucleophile to motivate the opening of the epoxide rings. The synergistic effect between the MOF catalyst and TBABr is therefore crucial in attaining high catalytic performance [93,94]. The cycloaddition reaction of CO_2_ with epoxides was extensively investigated using different potential catalysts [16,22,23,24,95,96,97,98,99,100,101,102].

**Figure 5:**
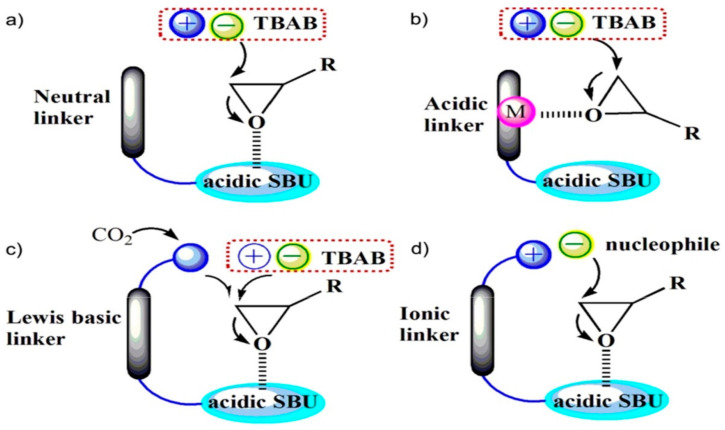
Epoxide activation modes by different MOF catalysts. (**a**) MOFs with acidic SBUs, (**b**) MOFs with acidic linkers, (**c**) MOFs with Lewis base linkers, (**d**) MOFs with ionic linkers. Reprinted with permission from Ref. [102]. Copyright 2019 Elsevier.

The best route for the mechanism was identified as the one that begins with the epoxides ring opening before the addition of carbon dioxide. This therefore proved that the catalytic system in this process strongly depends on the opening of the epoxide ring [19]. The different method for epoxide activation by the MOF catalyst was categorized into four bases on the features of the MOF catalyst (Figure 5) as follows: (a) MOFs with acidic secondary building units (SBUs) as the only active site, (b) MOFs with acidic linkers as metal active site catalyst, (c) MOFs with Lewis base linkers also acting as a nucleophile and Lewis acidic components, a binary catalytic system (d) MOFs with ionic linkers, where a single-component catalyst is used without the TBABr co-catalyst. The homogenous co-catalyst, tetrabutylammonium bromide (TBABr) would alone promote the epoxide ring-opening in (a and b) and the effort is reinforced in (c). The influence of Lewis-acidic component in SBUs or metal nodes of the MOFs, however, cannot be exempted as indicated (b–d) [102].

## 3. Metal-Organic Frameworks in CO_2_ Cycloaddition with Epoxides

**Figure 6:**
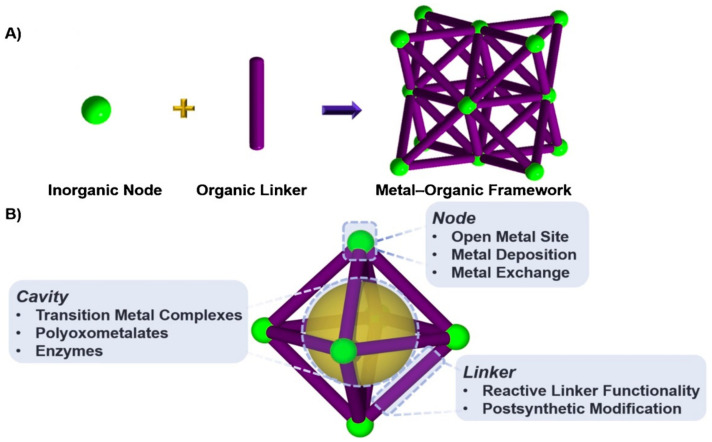
Special features of metal-organic frameworks: (**A**) Typical MOFs synthesis comprising inorganic nodes and organic linkers. (**B**) The accessibility of MOFs by modifying the node, linker, and content of the cavity. Reproduced with permission Ref. [107]. Copyright 2019 Elsevier.

**Figure 7:**
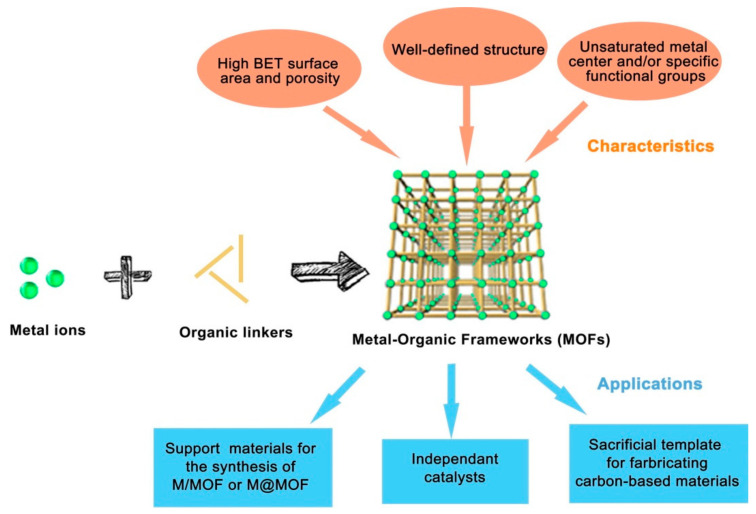
Illustration of MOF components, structure, characteristics, and mode of application. Reprinted with permission from Ref. [132]. Copyright 2018 Elsevier.

Metal-organic frameworks (MOFs) are a class of nanomaterials containing a cluster of metals and organic ligands (Figure 6) that attracted considerable attention because of their diverse topologies, tenability, and application in various fields (Figure 7) [103,104,105,106,107,108]. These nanoporous compounds have outstanding pore sizes of about 2 to 50 nm that have exhibited encouraging applications in adsorption [109,110,111,112,113,114], photocatalysis [115,116], and heterogeneous catalysis [8,87,117,118]. Different MOF materials were synthesized and employed as a catalyst in cycloaddition reaction of CO_2_ with epoxides and were shown to have reasonable potentiality in their applications [40,47,82,91,91,119,120,121,122,123,124,125,126,127,128,129,130]. The studies in some recent MOF materials employed as a catalyst in the formation of cyclic organic carbonates from CO_2_ and epoxides are summarized in Table 1.

Recent studies of some metal-organic framework materials as a catalyst for cycloaddition reaction (Table 1) reaffirmed that the cycloaddition reaction in most cases cannot proceed successfully without the presence of a co-catalyst. Tetra-n-butyl ammonium bromide (TBABr) was reported as the most effective co-catalyst among the various nucleophile components, TBAI, TBACl, and KI, and was identified to enhance epoxide ring opening in the reaction [120,131,132,133]. Some certain MOFs, however, were applied without the presence of a co-catalyst (Table 1, entry 17–20). Where this occured, the catalytic activity of MOFs were considered as a single component and were applied without the addition of TBABr. Nonetheless, based on the work studied, this type of reaction can only be successful under harsh reaction conditions of temperature and pressure (Table 1, entry 17–20) [55].

The addition of TBABr co-catalyst in most of the reported MOFs (Table 1, entry 1–16) further proved that the catalytic function of MOF catalysts in cycloaddition reaction works concurrently with the co-catalyst for successful conversion. Moreover, the catalyst/co-catalyst loading in the data entries (Table 1) was observed to be in relatively low percentage moles. The MOF catalytic materials were found to be active and were reused for at least three consecutive cycles in each case before losing their activity. All the reported MOFs (Table 1) were found to be effective at moderately ambient conditions, except for entry 17–20, which occured at relatively harsh conditions due to the absence of co-catalyst. Different epoxides such as propylene oxide, styrene oxide, and epichlorohydrin were found to undertake CO_2_ cycloaddition under the influence of the MOF catalyst, as shown in Table 1. 

**Table 1:** Recent studies of MOF catalysts in CO_2_ cycloaddition reaction to epoxides with reaction conditions.

Entry	MOF Material	Co-Catalyst	Catalyst: Cocatalyst Loading (mol%)	S_BET_ (m^2^/g)	Epoxide	Press (atm)	Temp. (°C)	Time (h)	Selectivity (%)	Yield (%)	Isosteric Heat Q_st_ (Kj/Mol)	Reusability	Reference
1	Al(OH) (O_2_C–CH=CH–CO_2_)∙*n*H_2_O	TBABr	0.02:0.002	1169	ECH	10	50	6	97	95	23	4 cycles	[134]
2	Zn_2_(Py)(Atz)_2_∙DMF∙2H_2_O	TBABr	0.1:0.1	764.5	PO	15	60	4	98	92	27.7	6 cycles	[118]
3	[In_2_(L)(OH)_2_]·2DMF·2H_2_O	TBABr	0.5:0.2	1022	EBH	1	70	12	89	99	-	5 cycles	[83]
4	F-Mn-MOF-74	TBABr	0.1:0.031	20.83	SO	10	100	6	99	99	-	7 cycles	[135]
5	PCN-222(Co)@MTTB	TBABr	0.1:0.216		PO/ECH	1	50	20	98	>98	-	3 cycles	[126]
6	rho-ZMOF	TBABr	0.1:1.4	871	ECH	10	40	3	98	98	-	5 cycles	[129]
7	Co-MOF-2 {[Co(BDC)(L)]·2H_2_O.xG}n	TBABr	1.8:2.5	6.8	SO/ECH	1	40	12	99	99	35.0	6 cycles	[119]
8	{[Zn(H_2_O)(HL)]⋅(DMF)_2_ (H_2_O)_2_}n	TBABr	0.25:0.232	945	PO	1	RT	48	-	76	-	-	[123]
9	MOF-5-MIX	TBABr	0.5:0.5	357	ECH	12	50	6	99	98	-	5 cycles	[63]
10	Ce-NU-1008	TBABr	0.02:0.002		SO	1	RT	20	95		-	3 cycles	[57]
11	Co-MOF-2 {[Co(BDC)(L)]·2H_2_O·xG}n	KI	5.0:0.2	6.8	SEO	1	40	8	99	99	35.0	6 cycles	[56]
12	{[Ni_3_HL(μ3-OH)(H_2_O)_2_]∙3(H_2_O)∙DMA}n	TBABr	0.025:1.5	743.5	ECH	10	100	6	-	>99	-	5 cycles	[33]
13	[(Cu_2_ BPDSDC∙4DMF)∙2DMF]n	TBABr	0.05:0.1	-	PO	25	80	5	98	99	-	4 cycles	[136]
14	{[Co_6_(OH)_2_(H_2_O)_4_ (cpt)_9_](NO_3_)(DMF)_13_}	TBABr	0.1:2	873	PO	1	40	48	97	97	32	4 cycles	[137]
15	InDCPN-Cl	TBABr	0.05:5.00	997	SO	1	80	24	98	93	30	5 cycles	[96]
16	Ce-NU-1008	TBABr	0.002:0.02	910	SO	1	RT	20	95		-	-	[57]
17	MOF-5@Imidazolium iodide	-	-	277.9	SO	10	110	8	-	92	-	4 cycles	[138]
18	[(CH_3_)_2_NH_2_][M(COOH)_3_]	-	13.1	13.11	PO	20	120	6	100	98	-	3 cycles	[139]
19	Im-MnF [C_3_H_5_N_2_][Mn(COOH)_3_]	-	-	81.57	ECH	15	100	6	99	95	-	-	[140]
20	Pt/Mg-MOF-74	-		513	PO	17.5	150	4	77	44	-	3 cycles	[72]

Note: All pressure units are converted to the approximate atm value.

## 4. MIL-101 Based MOFs in CO_2_ Cycloaddition with Epoxides

MIL-101 is one of the repeatedly reported MOF materials with a high potential catalytic activity for the conversion of CO_2_ to cyclic carbonates. This was ascribed to its possession of Lewis-acid sites due to Cr^2+^, present at the metal center [56] and structural flexibility, which allows its modifications by substituting different functional groups in the organic ligand but maintains the backbone structures. The synthesis and structural elucidation of MIL-101(Cr) was reported by different researchers [85,86,108,141,142,143,144,145,146,147,148,149].

**Figure 8:**
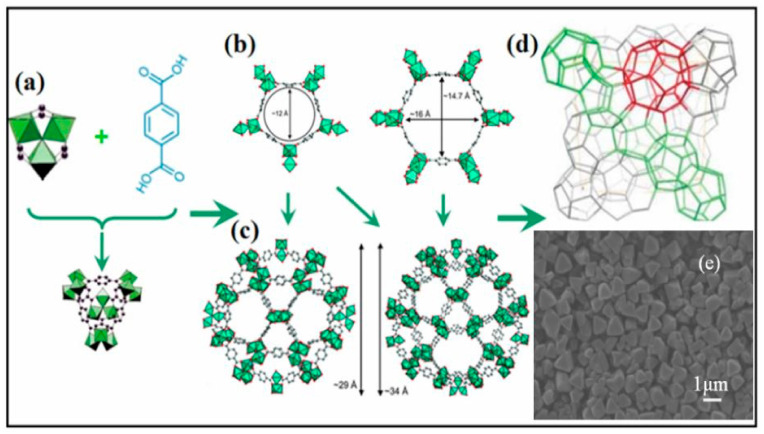
Synthesis and structural elucidation of MIL-101(Cr): (**a**) The formation of the hybrid super tetrahedron by the cluster chromium metal building unit and the bdc ligand; (**b**) Small pentagonal and larger hexagonal window (**c**) The two mesoporous cages; (**d**) 3D schematic representation of the MTN zeotype architecture (small cages are highlighted in green and large one is highlighted in red); (**e**) Morphology, showing clear octahedral shape with an average crystal size of ~1.0 μm [150].

**Figure 9:**
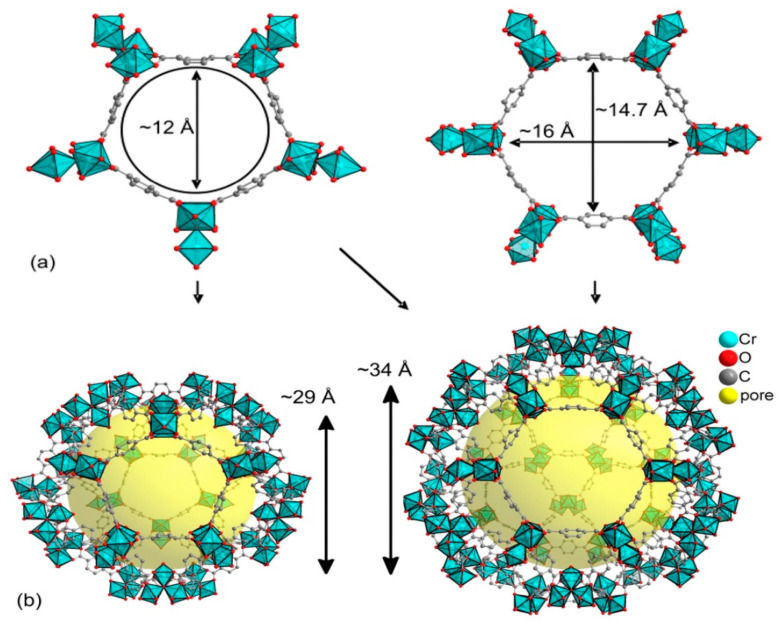
Trinuclear chromium building units and the bridging bdc ligands forming pentagonal and hexagonal rings (**a**) assembled into mesoporous cages (**b**) and mesoporous cages of yellow spheres with diameters of 29 or 34 Å, respectively. Reprinted with permission from Ref. [149]. Copyright 2016 Elsevier.

MIL-101 is a three-dimensional structure based on chromium terephthalate that was first synthesized by Fėrey et al. [148], having the empirical formula {Cr_3_(OH)(H_2_O)_2_O[(O_2_C)C_6_H_4_(CO_2_]_3_∙*n*H_2_O} with the given name MIL: (Materials Institute Lavosier) in 2005 (Figure 8). The material has a very stable structure with excellent water resistibility even under acidic conditions and was proven to have thermal stability up to 300 °C under air. The MIL-101(Cr) structure exhibited a large surface area of approximately 4100 m^2^ g^−1^ and contained two different types of cages with diameters of 29 and 34 Å, which had pore openings of 12 and 16 Å, respectively (Figure 9) [148,150,151]. Those special properties made MIL-101 possess superior catalytic activity, which was applied in different applications [110,111,152,153,154,155,156]. The unique porosity of three-dimensional frameworks forms exclusive channels with large surface areas, which can enhance CO_2_ by providing sufficient reaction spaces. It also allows the encapsulation of other catalytic active materials into the large pores to improve the catalytic activity of the MIL-101 by forming a composites with enhanced activity for application in various fields [143,155]. The MOF was also applied as a catalyst in cycloaddition reaction of CO_2_ with epoxides as a single component catalyst without the addition of co-catalyst [55].

We report here a series of recently reported MIL-101-based materials used as a catalyst in cycloaddition reaction of CO_2_ and epoxides.

Recently, research was reported by Akimana et al. [156], where MIL-101(Cr) was used to catalyzed cycloaddition reaction of epichlorohydrin to CO_2_ under mild reaction conditions without any co-catalyst. MIL-101(Cr) was identified as one of the catalytically active MOFs with excellent properties, chemical stability and flexibility, and strong acid sites due to the presence of metal centers in the framework [85,108,148]. It was gathered that special features of MIL-101(Cr) can facilitates the opening of the epoxide ring substrates in combination with the characteristic surface area and large pore size exhibited by the MOF, and that these are good properties for CO_2_ adsorption as well are essential features for a superior catalyst that can be applied in the formation of cyclic carbonates from CO_2_.

**Figure 10:**
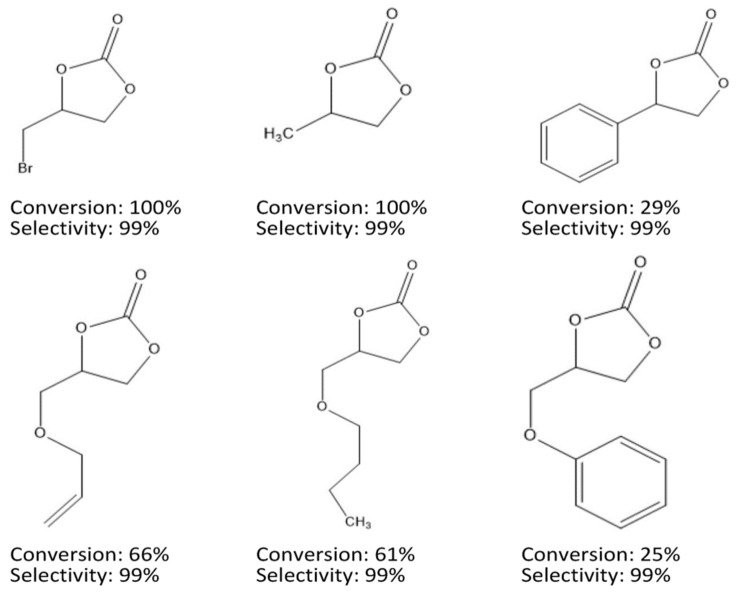
Organic carbonates obtained from MIL-101(Cr) catalyzed reaction. Reaction conditions: catalyst, 0.08 mmol; epoxide, 9.2 mmol; temperature, 35 °C; pressure, 1.5 bar [156].

The MOF was synthesized and applied to catalyze CO_2_ cycloaddition with different epoxides. The acidic or basic characteristic was provided by the metal nodes and the high porosity of the MOF attracts the CO_2_ gas molecules, which gave the MOF good ability to perform CO_2_ cycloaddition and produce cyclic carbonate compounds. The catalytic activity was observed by varying the conditions for the reaction using epichlorohydrin (ECH) as a model substrate. To obtain the optimum reaction condition, the various reaction conditions were alternated. Firstly by using a temperature of 35 °C and 4 bar CO_2_ pressure with no solvent or co-catalyst added (catalyst, 50 mg, ECH, 9.2 mmol). The outcome showed a 100% ECH conversion in 48 h. A CO_2_ pressure above 1.5 bar while maintaining the condition also exhibited an excellent performance of about 99% conversion in 24 h. The gap between conversion and yield was attributed to the MOF’s adsorption for the product and therefore was not detected in the H-NMR, resulting in low percentage yield. The conversion also decreased with a shorter reaction period or by reducing the catalyst loading amount. However, an average catalytic activity was observed with low catalytic loading under mild reaction conditions (35 °C, 1.5 bar) without the addition of co-catalyst or solvent [156]. The MIL-101(Cr) MOF catalytic activity for CO_2_ cycloaddition was also investigated with different epoxides and remarkable conversion was obtained Figure 10.

The catalyst shows amazing performance in catalytic activity when compared with other reported catalysts and has good reusability property. The application of MIL-101(Cr) as a heterogeneous catalyst in the conversion of CO_2_ into organic carbonates revealed high catalytic potentials that are greener and more feasible.

**Figure 11:**
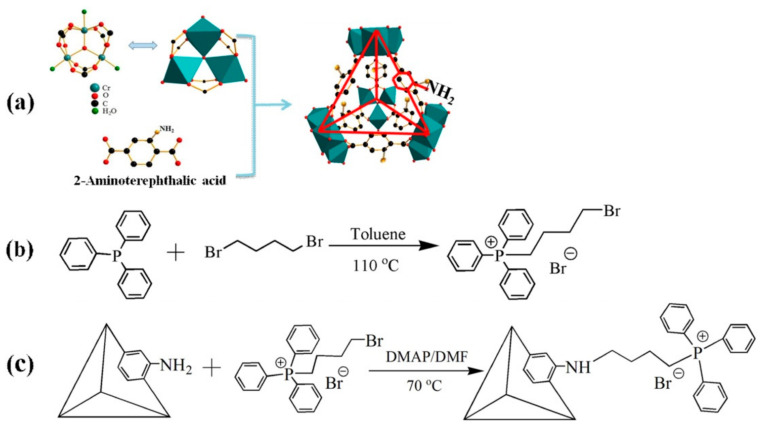
Preparation stages and model structure of Cr-MIL-101-[BuPh_3_P]Br. (**a**) Synthesis and activation of Cr-MIL-101-NH2, (**b**) Synthesis of (4-bromobutyl)triphenylphosphonium bromide, (**c**) Synthesis of [BuPh3P]Br-functionalized Cr-MIL-101. Reprinted with permission from Ref. [157]. Copyright 2020 Elsevier.

Dai et al. [157] recently synthesized a new MOF catalyst by implanting quaternary phosphonium salt ionic liquid (IL) on MIL-101(Cr) through a flippant post-synthetic modification to obtain (Cr-MIL-101-[BuPh_3_P]Br). The backbone structure of the parent MIL-101(Cr) was not altered when compared with the Cr-MIL-101-NH_2_, except for the broad Bragg reflections detected. The Cr-MIL-101-NH_2_ was then modified further by the addition of phosphonium salt IL through a covalent interaction between the –NH_2_ and R-Br group and the crystalline structure of the new hybrid was not affected by the post-synthetic modification (Figure 11). The presence of the phosphonium salt IL in the new compound was confirmed by XRD and FT-IR spectra. The new compound also showed more superior performance in its stability and catalytic activity when compared with the amine-functionalized MIL-101-IL reported earlier [158]. Due to their polarity, structural tenability, thermal stability, and excellent acid-basic property, ILs are considered a strong catalyst for CO_2_ cycloaddition reaction with epoxides. The fabrication of heterogeneous material containing active metal sites together with phosphonium salt IL can give rise to a good catalyst with excellent activity in cycloaddition reaction with epoxides [157].

**Figure 12:**
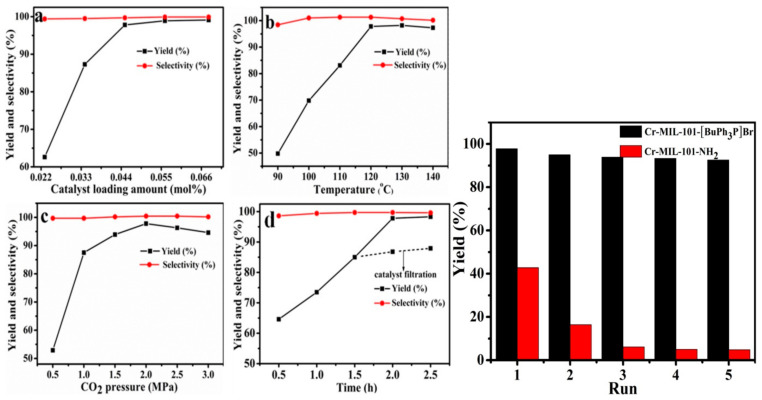
Effect of reaction parameters on the yield and selectivity (**left**) and recyclability test (**right**) under the same reaction condition. (**a**) temperature 120 °C, CO2 pressure 2.0 MPa, time 2 h; (**b**) catalyst 0.045 mol%, CO_2_ pressure 2.0 MPa, time 2 h; (**c**) catalyst 0.045 mol%, temperature 120 °C, time 2 h; (**d**) catalyst 0.045 mol%, temperature 120 °C, CO2 pressure 2.0 MPa. Reprinted with permission from Ref. [157]. Copyright 2020 Elsevier.

The catalytic function was utilized by the combined ability of two active sites involving Cr^3+^ as Lewis acid sites from the MOF and Br- as nucleophile from the IL, which facilitated the epoxide ring-opening by coordination of Cr^3+^ sites with an oxygen atom and nucleophilic attack of Br- on the carbon atom of epoxide, respectively. The catalyst performed an outstanding activity for the CO_2_ cycloaddition to propylene oxide under a moderate reaction condition of 120 °C and 2.0 Mpa for 2 h using a small amount of catalyst (0.045 mol%) without the addition of any solvent or co-catalyst (Figure 12). The reaction was found to be catalyzed by the individual sub-components of the catalyst, [BuPh_3_P]Br, Cr-MIL-101-NH_2_, Cr-MIL-101-[BuPh_3_P]Br, and Cr-MIL-101-NH_2_/[BuPh_3_P]Br, and each component were separately tested. It was observed that, although there was excellent selectivity in all cases, the conversion was relatively low in the case of [BuPh_3_P]Br and Cr-MIL-101-NH_2_ with only 24.3 and 42.8% yield, respectively. The highest yield was obtained by Cr-MIL-101-[BuPh_3_P]Br with 97.8% and turn over frequency (TOF) of 1087 [157].

An investigation was carried out to optimize the reaction parameters (catalyst loading, temperature, CO_2_ pressure, and time) and the outcome is illustrated in Figure 12. A sharp increase in the yield was observed by increasing the concentration of the catalyst, as shown in Figure 12a. The production of propylene carbonate (PC) indicated the need for sufficient energy in the reaction, as shown in Figure 12b, indicating the need for high temperature. The increase in pressure also indicated a significant effect on the reaction up to 2.0 MPa before it begins to have no positive effect on the reaction, as in Figure 12c. Finally, the reaction time showed a significant increase in the formation of PC from 0.5 h to almost 2.5 h (Figure 12d), and after that, extending the time did not affect PC production. Generally, the selectivity of PC was independent in all the parameters involved for the reaction and therefore did not have an effect. The recyclability test for both Cr-MIL-101-[BuPh_3_P]Br and Cr-MIL-101-NH_2_ was carried out in the research using similar reaction conditions and Cr-MIL-101-[BuPh_3_P]Br was reused more than four times without any noticeable reduction in PC production. The catalyst was also tested for the conversion of other epoxides and a significant yield was recorded [157].

**Figure 13:**
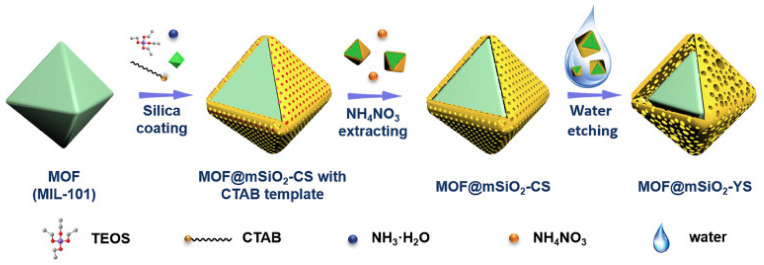
The schematic reaction for the formation of nanoreactors, MOF@mSiO_2_-YS; Step 1: MIL-101(Cr) encapsulation by SiO_2_ shell. Step 2: CTAB template removal by extraction give MIL-101@mSiO_2_-CS. Step 3: etching of MIL-101@mSiO_2_-CS using hot water. Reprinted with permission from Ref. [159]. Copyright 2020 Elsevier.

Bao et al. [159] reported a green synthesis and cost-effective method for yolk-shell structures of water etching of MOF. The research was carried to fabricate a MOF@mSiO_2_ as yolk-shell nanoreactors by silica coating method, which was followed by a water-etching approach. The material was fabricated to curtail the stability challenges of MOF catalyst under hydrothermal conditions as a result of weak metal-ligand bonds in their structures. In this regard, a high-density Lewis acid MIL-101 having unique structures and many catalytic advantages, especially for the acid catalytic reaction, was selected to be encapsulated into SiO_2_ and make a yolk-shell arrangement by engraving the MIL-101(Cr) surfaces with water, as shown in Figure 13. The structure of the nanoreactors MIL-101@mSiO_2_-YS was studied and characterized by various spectroscopic techniques, which proved the successful formation of MIL-101(Cr)@mSiO_2_-YS.

**Figure 14:**
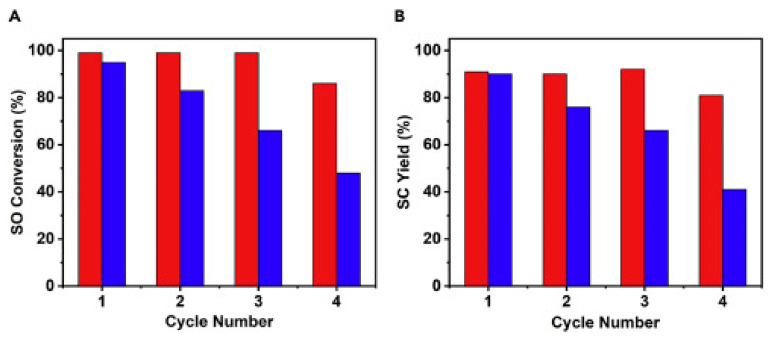
Catalytic performance of MIL-101 (blue) and MOF@mSiO_2_-YS (red) for cycloaddition reaction of SO with CO_2_: (**A**) SO conversion, (**B**) SC yield. Reprinted with permission from Ref. [159]. Copyright 2020 Elsevier.

The study of the new structure revealed that the MIL-101(Cr) nanostructure will have the ability to maintain its Lewis acid sites and the nanoshell structure will deliver effective paths for bulk movement of reactants and products, while the yolk-shell safeguards the MIL-101 and reduces its disintegration during catalytic reaction processes. Thus, the combined special effects of the MIL-101(Cr) framework and that of SiO_2_ yolk shells are perfectly brought together to accomplish superior properties and stability that can be used in CO_2_ cycloaddition reaction. A cycloaddition of CO_2_ with styrene oxide (SO) was tested using the prepared composites and the outcome showed improved catalytic activity by having more cycles compared to the pristine MIL-101, which began to decrease in its catalytic activity after three cycles by the decrease in the yield of styrene carbonate from 98 to 66% due to collapse in its structure, as in Figure 14. The CO_2_ reaction was carried in the presence of TBABr co-catalyst using SO in an autoclave charged with 0.22 mmol catalysts; MIL-101@mSiO_2_-YS, 18 mmol SO, 0.62 mmol TBABr, and 0.8 MPa CO_2_ pressure under stirring for 48 h at room temperature [159].

**Figure 15:**
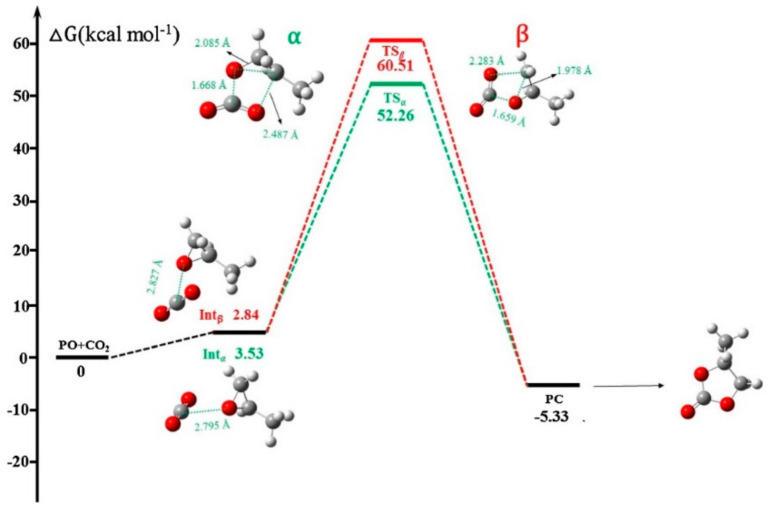
Gibbs free energy surface profile in a non-catalyzed reaction with optimized geometry of intermediates and transition state. Reprinted with permission from Ref. [67]. Copyright 2018 Elsevier.

**Figure 16:**
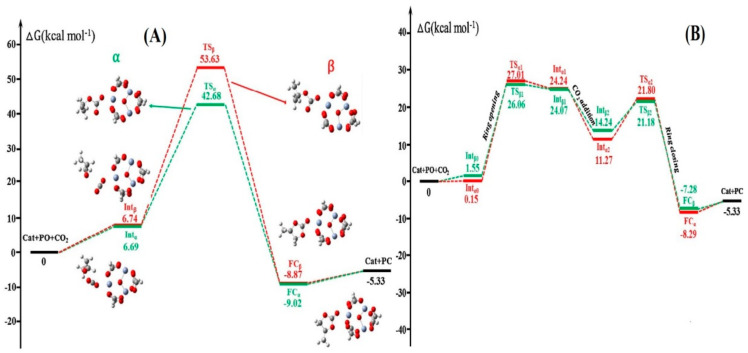
Gibbs free energy surface profile of (**A**) MIL-101-catalyzed reaction (**B**) TBABr-catalyzed reaction, with optimized geometries of intermediates and transition states. Reproduced with permission Ref. [67]. Copyright 2018 Elsevier.

Hu et al. [67] reported a DFT study on the cycloaddition of CO_2_ to propylene oxide (PO) using binary system MIL-101/TBABr catalyst. A comparative study of non-catalyzed reaction Figure 15, MIL-101-catalyzed reaction as a single component catalyst (Figure 16A) and TBAB-catalyzed reactions (Figure 16B) were analyzed using Gibbs free energy surface profile and the transition states in the conversion processes were illustrated. It was discovered that the reaction in the binary system MIL-101/TBABr proceeded in a much simpler manner with a lower energy barrier (18.11 kcal mol^−1^) and 90% conversion. On the other hand, the MIL-101-catalysed alone reaction required a minimum energy of (46.89 kcal mol^−1^) and the TBABr-catalyzed alone required an energy of (26.86 kcal mol^−1^) with the conversion of 27 and 42%, respectively, while the non-catalyzed reaction, which proceeded under ambient conditions, required a minimum energy of (57.67 kcal mol^−1^) [67].

**Figure 17:**
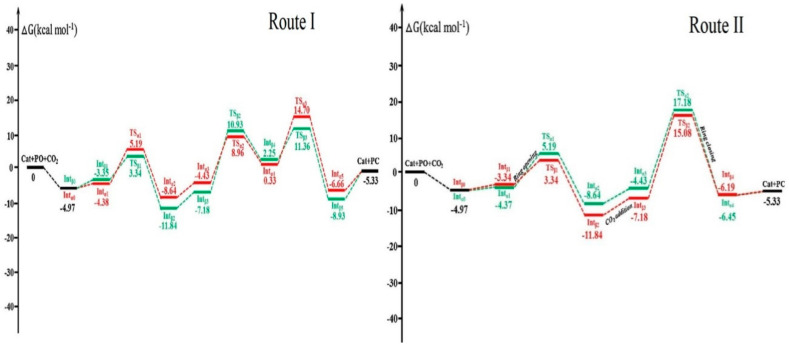
Gibbs free energy surface profiles in CO_2_ cycloaddition of PO catalyzed by MIL-101(Cr)/TBABr. Reprinted with permission from Ref. [67]. Copyright 2018 Elsevier.

The mechanism study for the reaction showed a three-step path involving (i) the epoxide ring-opening, (ii) the carbon dioxide addition, and (iii) the ring-closing of the cyclic carbonate was preferentially established and was more kinetically positive compared to a two-step path, involving the opening of the epoxide ring and closure of the cyclic carbonate ring only. The Gibbs free energy surface profile for the two different routes is illustrated in Figure 17. The outcomes of the computational studies affirmed that in the presence of a binary catalyst (MIL-101/TBABr) the cycloadding of CO_2_ to PO occurs more easily compared to both non-catalyzed and TBABr-Catalyzed and MIL-101(Cr)-catalyzed pathways [67].

**Figure 18:**
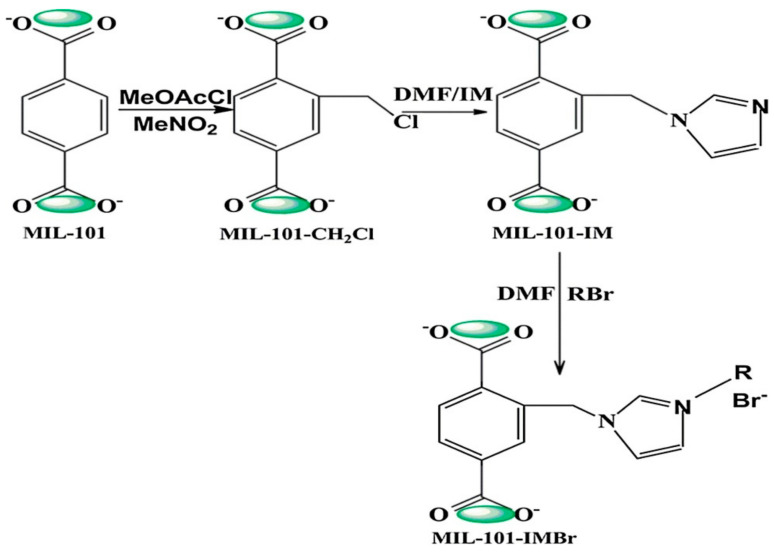
Schematic representation for the synthesis of MIL-101-IMBr. Reprinted with permission from Ref. [160]. Copyright 2018 Elsevier.

**Figure 19:**
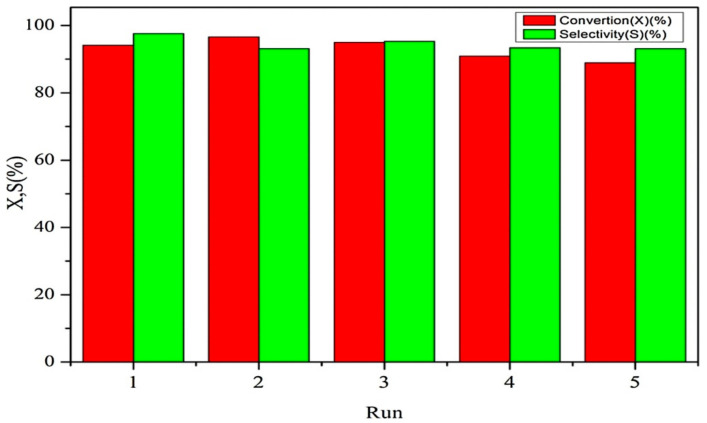
Reusability studies of MIL-101-IMBr-6. Reproduced with permission Ref. [160]. Copyright 2018 Elsevier.

Liu et al. [160] carried an investigation for the coupling of propylene oxide (PO) with CO_2_ using MIL-101-based MOF composites prepared by post-synthesis and modification of MIL-101(Cr) as Lewis acid site with imidazolium-based ionic liquids to obtained MIL-101-IMBr composite, which fully characterized using different spectroscopic techniques, as in Figure 18. The ionic liquid was functionalized with basic sites that serve as a nucleophile in the reaction by providing halides ions to motivate the opening of the epoxide ring in the cycloaddition reaction. These special features made the composite possess a good catalytic property for cycloaddition reaction at moderately mild condition (80 °C and 0.8 MPa) without the addition of co-catalyst. The PO conversion reached more than 95% with 97% selectivity of propylene carbonate (PC). The catalyst also showed excellent reusability (Figure 19) and had no visible defect in the catalytic activity throughout the circles [160].

**Figure 20:**
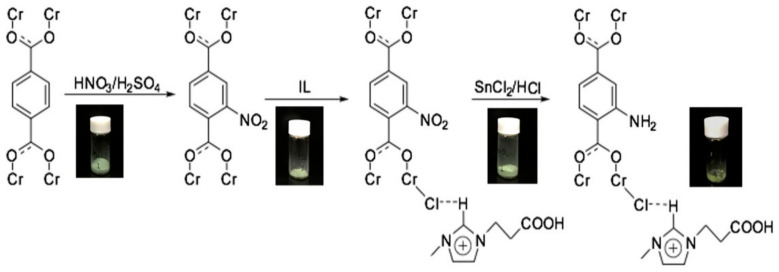
Preparation stages for the synthesis of IL/MIL-101-NH_2_. Reprinted with permission from Ref. [158]. Copyright 2018 Elsevier.

Wang, et al. [158] reported similar research by immobilizing ionic liquid (IL) on an amine-functionalized metal-organic framework in their effort to synthesize bifunctional catalyst with acid-base property for application in CO_2_ cycloaddition reaction. The immobilization between the IL and MIL-101-NH_2_ was constructed via a post-synthetic procedure to incorporate both amino and carboxylic groups into the material, as shown in Figure 20. The study was supported by density functional theory (DFT) calculations and the hybrid MOF was found to possess acid-base bifunctional futures, and was used as a single catalyst without co-catalyst addition.

The synthesized hybrid MOF (IL/MIL-101-NH_2_) was applied as a catalyst in the formation of propylene carbonate from CO_2_. The reaction was carried out in a low pressure of CO_2_ of 1.3 MPa and 1 h duration at a temperature of 120 °C, resulting in 91% yield. The strategic design and synthesis of the catalyst with characteristic acid-base properties opened a new window in the development of new material that can catalyze cycloaddition reaction of epoxides to CO_2_. The composite was found to be very stable by recycling up to five times without a change in its structure [158].

## 5. HKUST-1 for CO_2_ Cycloaddition with Epoxides

**Figure 21:**
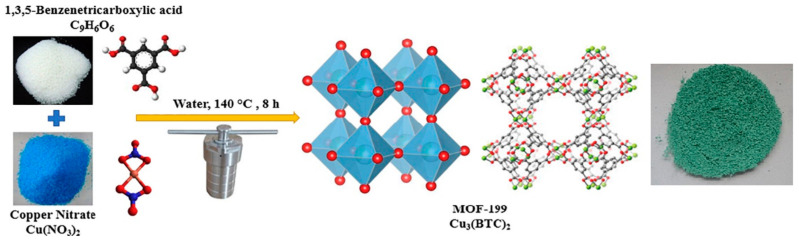
Schematic Procedure of HKUST-1 synthesis and structural elucidation. Reprinted with permission from Ref. [161]. Copyright 2019 Elsevier.

**Figure 22:**
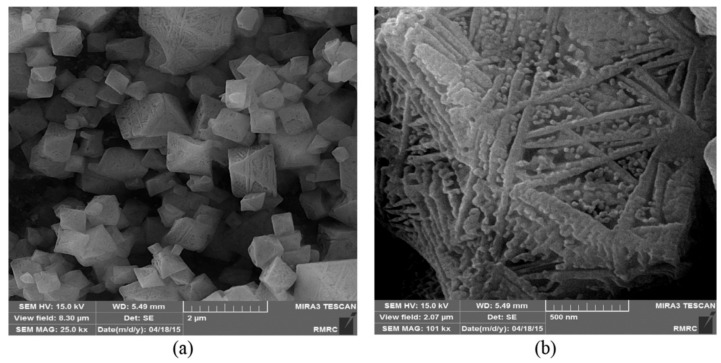
FESEM micrographs of HKUST-1 in different scales: (**a**) 2 μm, (**b**) 500 μm. Reprinted with permission from Ref. [161]. Copyright 2019 Elsevier.

**Figure 23:**
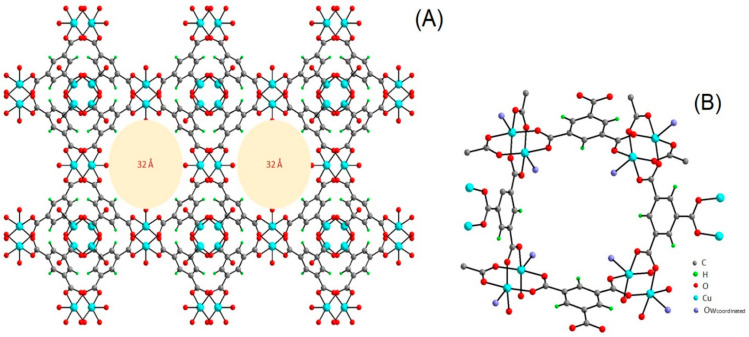
(**A**) Structure of HKUST-1. (**B**) Paddle-wheel secondary building unit (SBU). Reprinted with permission from Ref. [58]. Copyright 2019 Elsevier.

Another group of porous material that attracted the attention of researchers as a potential catalyst for CO_2_ cycloaddition with epoxides is HKUST-1 (Hong Kong University of Science and Technology) and also referred as MOF-199, a Cu-based of 1,3,5-benzenetricarboxylate (BTC) ligand. HKUST-1 was first synthesized in 1999 using copper(II) ion (Cu^2+^) as metal center and BTC ligand to give a light blue powder (Figure 21) [87,88,161], it was also reported using other metals with +2 oxidation state such as Cr^2+^ [162], Ni^2+^ [163], Zn^2+^ [164], Fe^2+^, Co^2+^ [165,166], and as bimetallic with two different metals at the center while maintaining the structure [167]. HKUST-1 material has a molecular formula [Cu_3_(BTC)_2_(H_2_O)_3_] and morphological structure with a sound level of thermal stability (Figure 22) and a capacity for chemical functionalization of the channel linings with face-centered-cubic crystals comprising a 3D intersecting system with big square-shaped pores (9 Å by 9 Å) of a continuous network through Cu(OAc)_4_ paddle wheel SBU, as in Figure 23. The open metal sites in the HKUST-1 structure provides the basis for CO_2_ adsorption property [7,168]. The large porous structure can also be used to create effective heterogeneous catalysts as templates to construct dynamic heterogeneous system by encapsulation of other nanomaterials to form composites. The resultant composite material can preserve the original properties of the HKUST-1, while gaining additional unique properties that can be applied in different applications [104,169,170,171,172].

In recent years, progress was made by various researchers on the modification and application of HKUST-1-based MOFs as a potential catalyst for CO_2_ cycloaddition reaction with epoxides. Some of these findings are summarized here.

**Figure 24:**
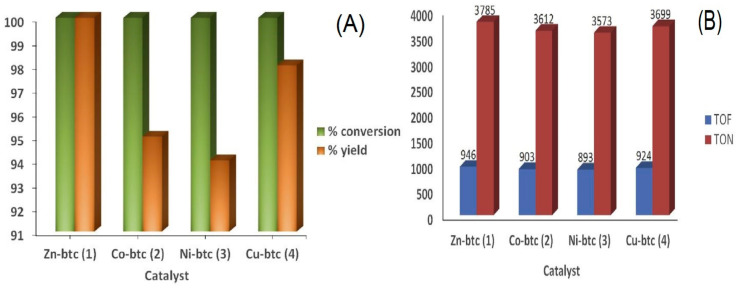
(**A**) The conversion (%) and yield (%) (**B**) TON and TOF: for cycloaddition conversion of CO_2_ to PC using M-btc MOFs (M = Zn, Co, Ni, and Cu). Reaction condition: temperature, 120 °C; CO_2_ pressure, 0.7 MPa; time, 4 h. Reprinted with permission from Ref. [58]. Copyright 2019 Elsevier.

A series of benzene tricarboxylic acid (btc)-based metal-organic frameworks were synthesized by Rani, et al. [58]. The MOFs were solvothermally synthesized under the same experimental conditions and fully characterized by different analytical methods. The synthesized MOFs included; Zn, Co, Ni, and Cu btc-based MOFs, which were described to exhibit diverse structural and catalytic behaviors [58]. A study on the application of MOFs for CO_2_ cycloaddition with PO under mild conditions was carried out successfully. For the first time, Zn, Co, and Ni btc-based frameworks were exploited as a catalyst for CO_2_ cycloaddition reaction with epoxides and displayed remarkable yield under certain reaction conditions. An attempt was made previously with Cu-based (HKUST-1) MOF on this catalytic application, but interestingly, in this study, the Cu-btc was also reported to exhibit higher yield than in the previous reports [54,169]. The research further revealed that among the four synthesized MOF catalysts, Zn-btc was identified to perform higher catalytic performance in the CO_2_ fixation reaction as compared with other MOFs having a large turnover number (TON) of 3785 mol^−1^ and a turnover frequency (TOF) of 946 h^−1^ with more than 99% yield (Figure 24) [58].

**Figure 25:**
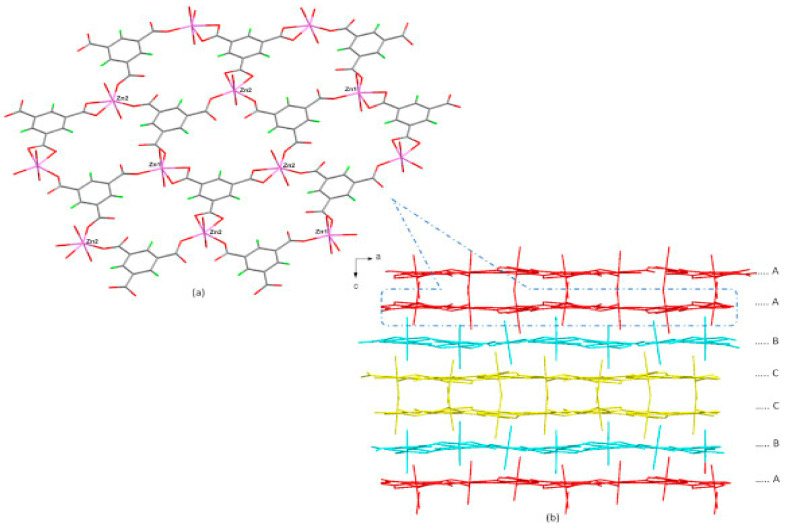
(**a**) A 2D sheet framework of Zn-btc (**b**) stacking of the 2D sheets over one another, through C-H ….O bonding. Reprinted with permission from Ref. [58]. Copyright 2019 Elsevier.

The structural features of the synthesized MOFs were critically analyzed to further reveal the chemistry relating to their high catalytic performance. The white crystals of Zn_3_(btc)_3_(H_2_O)_6_∙2H_2_O were obtained by reacting zinc nitrate salt with 1,3,5-benzene tricarboxylic acid under solvothermal conditions at a temperature of 85 °C for 24 h. The Zn_3_(btc)_3_(H_2_O)_6_∙2H_2_O, (Zn-btc), formed a hexagonal white crystal with the P6_5_22 space group as revealed by XRD analysis. The Zn-btc asymmetric unit contained [Zn_2_(btc)_2_(H_2_O)_4_] and two molecules of lattice vapor. In the asymmetric unit, there were two types of Zn atoms; each was present in a distorted octahedral geometry and linked in the planar position to three separate btc ligands and two coordinated water molecules in the equatorial position. Two among the three btc ligands acted as bidentate ligands. The Zn_2_-coordinated bidentate ligand was tentatively coordinated with additional Zn_1_ and Zn_2_. The overall coordination of Zn and btc ligands contributed to the creation of 2D sheet structure, as shown in Figure 25 [58].

**Figure 26:**
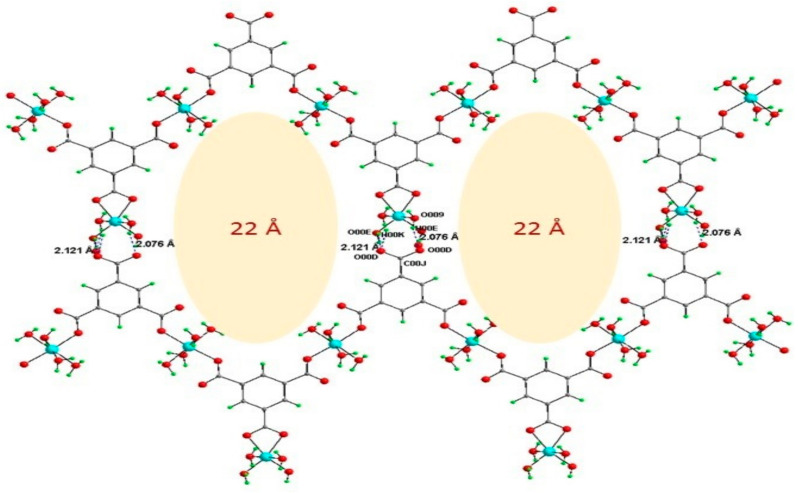
Zig-zag chains in [M_3_(*btc*)_2_(H_2_O)_12_] [M = Ni, and Co] are connected via H-bonding forming a 2D sheet with void space of approximately 22 Å. Reprinted with permission from Ref. [58]. Copyright 2019 Elsevier.

**Figure 27:**
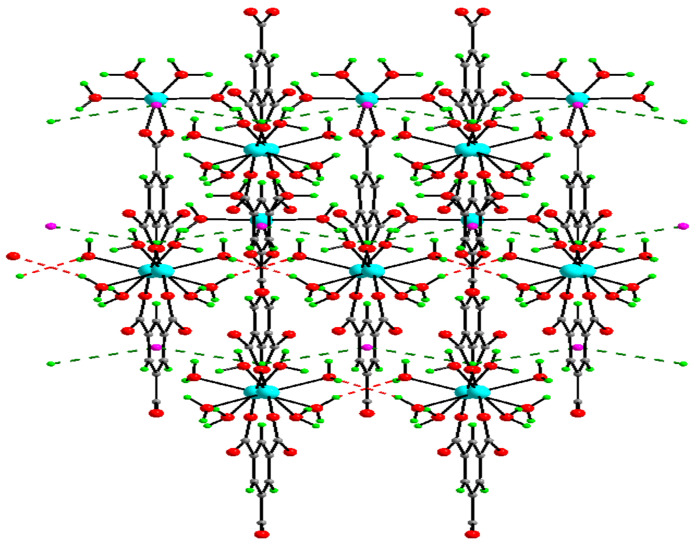
3D framework in [M_3_(*btc*)_2_(H_2_O)_12_] [M = Ni and Co]. Reprinted with permission from Ref. [58]. Copyright 2019 Elsevier.

The Co-btc and Ni-btc crystals were obtained using the same experimental conditions by reacting cobalt nitrate and nickel nitrate with btc ligand, respectively. SXRD was used to interpret the structures of the two MOFs, which contain two distinct metal centers that are isostructural, both in a distorted octahedral geometry. The structure consisted of coordinated metal centers with two molecules in the central position and two water molecules and btc acting as a bidentate ligand in a planer position. Another metal center was coordinated to two monodentate btc ligands in the central position and four water molecules in a planar position. The btc was bidentatively connected on both sides to one metal center and monodentatively to two separate metal centers and vice-versa, forming a zig-zag ID chain through H-bonding, which resulted in forming a 2D-sheet structure with large pores (Figure 26). The resulting structures were reconnected via C-H π bond and O-H weak bond interaction to form 3D structure (Figure 27) [58].

A single crystal structure study for Cu-btc showed the Cu metal in a square pyramidal geometry coordinated with four planar btc carboxylic oxygen molecules and water molecules in an equatorial position. Via four btc ligands, two Cu centers were connected forming a structure resembling paddle wheels, which were interconnected to give a 3D system structure with large pores of ~32 Å as earlier described, see Figure 23.

The recyclability test for all the synthesized MOF catalysts was investigated using the optimum conditions at 120 °C and CO_2_ pressure of 0.7 MPa for the length of 4 h to explore the catalyst recycling ability of the MOFs. The outcome reveals that Zn-btc had the highest recycling ability, followed by Cu-btc. [58]

Another related research was carried out by Ding et al. [133] who conducted a computational study on a series of HKUST-1(Cu-btc) structures with different metal centers to design a special catalyst for CO_2_ fixation with epoxides. The catalytic activity of M-HKUST-1(M = Sc, Ti, V, Cr, Mo, Cd, Ru, W, Cu, Fe, Zn) under the influence of TBA salts in a series of four different halogen anions denoted as TBAX (X = F, Cl, Br and I) as co-catalysts in the formation of propylene carbonate from CO_2_ and PO was investigated. The recent development in computing technology has driven great advancement in designing materials with high accuracy and efficiency and this technology was employed in the development of new catalytic materials for the fixation of CO_2_, which mainly depends on trials of a different experiment.

**Figure 28:**
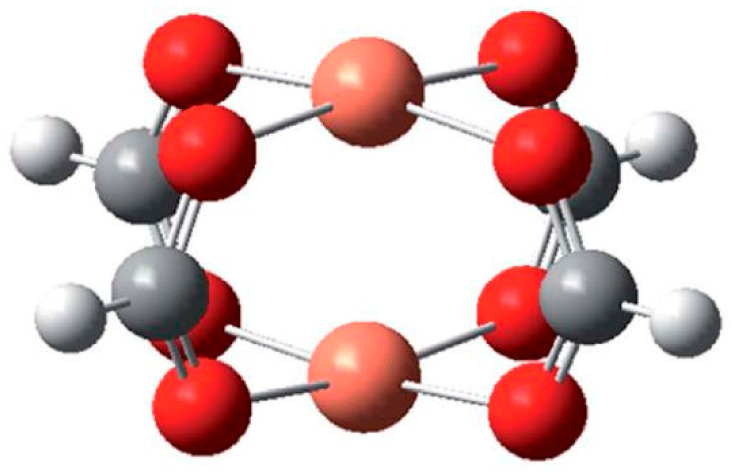
Cluster model used for the density functional theory (DFT) calculations of M-HKUST-1. Reprinted with permission from Ref. [133]. Copyright 2019 Royal Society of Chemistry.

The models for Cr-HKUST-1, Cu-HKUST-1, and Fe-HKUST-1 obtained from the Crystallographic Data Centre (CCDC) of Cambridge University and others through isostructural substitution of the Cu atom having identical crystal environment from the known HKUST-1 MOF. A cluster model with a narrow dimension was utilized for the single-site catalytic reaction, which usually happens inside the HKUST-1 cavities. The study of the mechanism was carried out by using condensed cluster models and the effect of the metal substitution was further studied. The simple cluster was developed to imitate the HKUST-1 framework (Figure 28).

**Figure 29:**
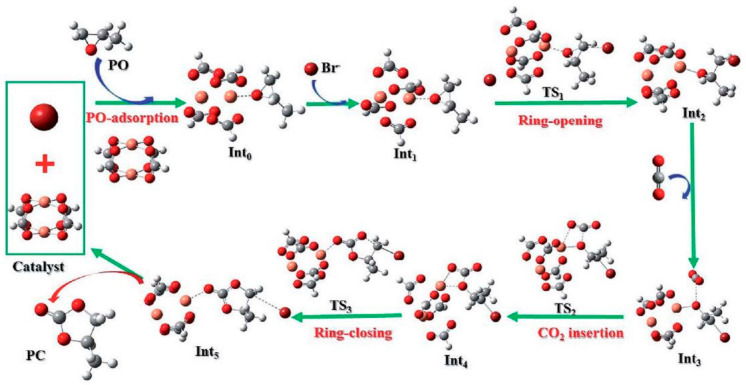
The mechanism study for the reaction of CO_2_ with PO to obtained cyclic carbonates catalyzed by M-HKUST-1. Reprinted with permission from Ref. [133]. Copyright 2019 Royal Society of Chemistry.

**Figure 30:**
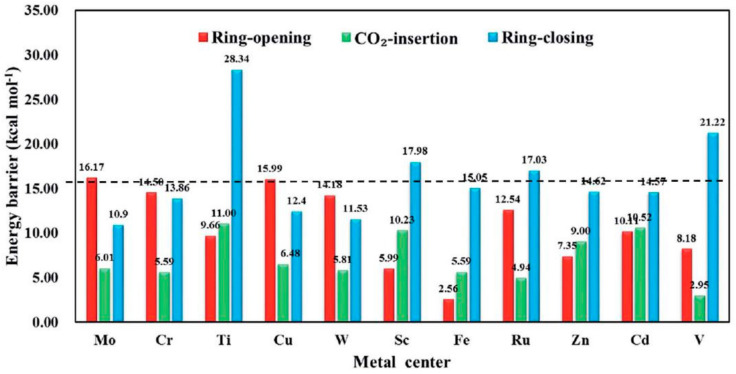
Gibbs energy barrier for CO_2_ cycloaddition with PO using M-HKUST-1 as a catalyst. Reprinted with permission from Ref. [133]. Copyright 2019 Royal Society of Chemistry.

The most generally accepted mechanism for a MOF-based catalyst in CO_2_ cycloaddition with epoxides can be summarized into four steps, starting with the adsorption of epoxide, epoxide ring opening, CO_2_ insertion, and finally cyclic carbonate formation by closing the ring. The predicted mechanism for HKUST-1 is illustrated in Figure 29. The study also revealed a similar mechanism to that of regular Cu-HKUST-1 with the ring-opening playing the most critical part, not the insertion of CO_2_. This was calculated to require moderately lower barrier as defected in the Gibbs free energy barrier (Figure 30) [133].

The M-HKUST-1 computational screening can be depicted by studying the rate-determining step in the reaction. This can be obtained by assessing the activity of the whole M-HKUST-1 systems, thereby discovering the most effective catalyst. The energy barriers for the key step were prescribed as presented in Figure 30. For the M-HKUST-1/Br^−^ catalyst, the rate-determining energy barrier relied primarily on the metal centers. According to the studies, W-HKUST-1 would accomplish a high potential to attain superior activity among the M-HKUST-1 system. Furthermore, TBABr was forecast as the most effective co-catalyst among the TBAX studied for the better catalytic function of M-HKUST-1 [133].

Wu et al. [17] carried a similar analysis in 2018 on btc-based MOF using different metal centers (M = Mn, Ni, Co) with a general formula, [(CH_3_)_2_NH_2_][M_3_(BTC)(HCOO)_4_(H_2_O)] H_2_O denoted as M-BTC. The MOF was prepared and used under various reaction conditions to catalyzed CO_2_ cycloaddition with epichlorohydrin (ECH). The catalysts were fully characterized using different analytical methods and highest catalytic activity was recorded in Mn-BTC with over 98% conversions of ECH and 96% selectivity at a temperature of 105 °C, 3.0 MPa CO_2_ pressure for 9 h. Mn-BTC catalyst also showed remarkable stability and could be reused with a small reduction in its catalytic ability over three times. Moreover, CO_2_ was added to other epoxides and DFT calculations were performed, and the yields revealed in the order ECH > propene oxide > 1,2-epoxybutane > allyl glycidyl ether [17].

Previously, research was carried out by Kurisingal et al. on two forms of metal-organic frameworks (Cu-BTC and UiO-66) as a binary system, which were synthesized solvothermally for the first time and applied as catalysts for CO_2_-epoxide cycloaddition without the addition of solvent. The research focused on binary MOF with Cu and Zr metal centers and assessed their catalytic potentials for CO_2_ conversion into cyclic carbonate compounds with epoxides in the absence of any solvent. The study of the effects of some reaction parameters such as catalyst amount, temperature, reaction time, and CO_2_ pressure was examined. The synthesized binary MOFs were used for the CO_2_-epoxide cycloaddition in the presence of TBABr co-catalyst. The catalyst (UiO-66/Cu-BTC) accomplished good conversion for epichlorohydrin (ECH) with over 99% selectivity. The remarkable conversion of ECH by the UiO-66/Cu-BTC/TBAB binary system was promoted by the combined effect of the two metal centers (Cu and Zr) and the bromide ion (Br^−^) from TBAB. The catalyst reusability was explored by reusing the catalyst six times without losing its catalytic properties [64].

## 6. Summary and Outlooks

The application of metal-organic framework-based catalytic materials for the CO_2_ cycloaddition with epoxides in some recent studies was summarized and reported in this review. MIL-101(Cr) and HKUST-1 based MOFs were identified as a high potential MOF catalysts for the conversion of CO_2_ into cyclic organic carbonates. The two forms of MOF materials, MIL-101(Cr) and HKUST-1, were both identified to possess special features that gave the advantage in catalyzing the reaction of CO_2_ with epoxides. Both MIL-101(Cr) and HKUST-1 frame works were characterized with Lewis-acid property due to the presence of metal (II) ions at their metal centers and their structural flexibilities. Thus, allowing their modifications by replacing different functional groups in the organic ligand of MIL-101 and substituting the metal centers in HKUST-1 respectively, while maintaining their backbone structures. The large porosity in the structures of the two materials contributed to their adsorption ability in CO_2_ and can also be utilized in making effective heterogeneous system by encapsulation of other nanomaterials to form composites. The resultant composites would preserve the original properties of the MOFs, while gaining additional unique properties that can be applied in different applications. There is currently on-going research to improve the catalytic properties of the two MOFs (MIL-101(Cr) and HKUST-1) for better performance. The review also shown that MOF-based catalysts operate effectively when coupled with a co-catalyst. The presence of a co-catalyst helps in performing the crucial role of the activation of the epoxide ring-opening in the second step of the reaction chain. Among the several co-catalyst materials, the one with high electrophilic ability served the best in catalyzing the reaction and for this process, tetrabutylammonium bromide (TBABr) was proven to show the highest performance. The continuous modification of the porous MOFs revealed positive results towards increasing their catalytic activity for CO_2_ cycloaddition with epoxides.

## Data Availability

Not applicable.

## Abbreviations

MOFs	Metal-organic frameworks
TBABr	Tetrabutyl ammonium bromide
SBU	Secondary Building Unit
SO	Styrene Oxide
PO	Propylene oxide
PC	propylene Carbonate
ECH	Epichlorohydrin
EBH	Epibromohydrin
SEO	Spiro-Epoxy Oxindole
BPE	1,2-bis(4-pyridyl)ethylene ligand
BTC	1,3,5-benzenetricarboxylate
HKUST	Hong Kong University of Science and Technology
RT	Room temperature
KI	potassium iodide
Å	Aperture
CTAB	Cetyltrimethylammonium bromide
